# Batch correction methods used in single-cell RNA sequencing analyses are often poorly calibrated

**DOI:** 10.1101/gr.279886.124

**Published:** 2025-08

**Authors:** Sindri Emmanúel Antonsson, Páll Melsted

**Affiliations:** Faculty of Industrial Engineering, Mechanical Engineering, and Computer Science, University of Iceland, 102 Reykjavík, Iceland

## Abstract

As the number of experiments that employ single-cell RNA sequencing (scRNA-seq) grows, it opens up the possibility of combining results across experiments or processing cells from the same experiment assayed in separate sequencing runs. The gain in the number of cells that can be compared comes at the cost of batch effects that may be present. Several methods have been proposed to combat this for scRNA-seq data sets. We compare eight widely used methods used for batch correction of scRNA-seq data sets. We present a novel approach to measure the degree to which the methods alter the data in the process of batch correction, both at the fine scale, comparing distances between cells, as well as measuring effects observed across clusters of cells. We demonstrate that many of the published methods are poorly calibrated in the sense that the process of correction creates measurable artifacts in the data. In particular, MNN, SCVI, and LIGER perform poorly in our tests, often altering the data considerably. Batch correction with Combat, ComBat-seq, BBKNN, and Seurat introduces artifacts that could be detected in our setup. However, we find that Harmony is the only method that consistently performs well in all the testing methodology we present. Therefore, Harmony is the only method we recommend using when performing batch correction of scRNA-seq data.

With the increasing complexity and scale of scRNA-seq experiments, the need to integrate and combine differently collected data has also increased. Large scale data sets enable researchers to combine and compare different data modalities to gain increased insight and accuracy. When scRNA-seq data are collected at different times, with different protocols, technologies, or sequencing platforms, the integration becomes increasingly complex. All these factors can affect the expressions of genes in complex ways; some of these differences can be biological in origin, others from technical artifacts. We aggregate the variation due to technical artifacts under the umbrella term of batch effects.

There are some unique challenges in integrating batches of scRNA-seq data that are not present when working with bulk RNA-seq data. Cell type composition can differ between batches, and within cell types, there can be systematic differences in gene expression between batches (Luecken and Theis 2019). One of the first steps in processing scRNA-seq data is to cluster or identify cells by cell type, thus requiring batch correction methods tailored for scRNA-seq data sets in order to ensure that cells of the same type are grouped together across batches.

Other studies that have evaluated the performance of batch correction methods focus more on measuring how well batch effects are removed rather than how well the variation of interest is retained or whether the methods are well calibrated. Early work (Tran et al. 2020) compared 14 different batch correction methods based on run time and classical metrics from cluster comparison. A recent preprint (Tyler et al. 2021) approached the problem of batch correction from both viewpoints, evaluating how well batch effects are corrected and the extent to which real biological effects are preserved after batch correction, demonstrating a certain level of erasure of biological signal with unsupervised batch correction methods and that steps should be taken to mitigate that. Overall, their results are consistent with the results presented in this paper. The task of atlas-level integration was considered in Luecken et al. (2022), who found that methods such as LIGER, BBKNN, and Seurat v3 tended to favor removal of batch effects over conservation of biological variation, and that, for a particular type of data, Harmony could integrate strong batch effects while retaining biological variation in some cases.

Any systematic effects on gene expression, affecting a large number of genes, will affect each point of the computational pipeline, that starts with the raw sequencing data or count matrix and ends with a statistical test that is computed in order to demonstrate a biological difference. This systematic bias must be addressed, regardless of whether the effect is biological in origin (e.g., cells from different environments or individuals) or technical (i.e., the result of batch effects).

Although most of the proposed methods have demonstrated, in their respective manuscripts, that they are capable of removing batch effects, they do not show that, in the absence of batch effects, the batch “corrections” do not alter the underlying truth.

We propose a methodology to assess the quality of these methods and to measure the extent to which these methods alter the data in the process of batch correction—in particular, the case when there is little or no batch effect. We perform the batch correction and examine the change, if any, the process of each batch correction method has on the data and all downstream results.

Ideally, the application of batch effect correction should not correct the data at all as measured by a statistical test—that is, the methods should be well calibrated. Under this null hypothesis, any significant change can be classified as an artifact of batch correction.

We present different data sets and use them to establish a ground truth: the distances between cells and the *k*-nearest neighbor (*k*-NN) structure, etc. We then split them into two equal parts to create pseudobatches.

We examine the effects of the batch correction on three representative stages of the most common computational pipelines in scRNA-seq analysis: (1) the *k*-NN graph computed from the count matrix; (2) clustering and cell type identification; and (3) differential expression analysis between clusters. We evaluated eight computational methods for batch correction that are commonly used in scRNA-seq pipelines.

## Results

### Batch correction methods

We selected eight different batch correction methods for evaluation, based on how commonly they are used and the difference in their respective methodologies. The selection criteria were primarily based on either good performance in previous evaluations, a large number of uses in the literature, or if they are included in analysis frameworks.

A recent paper (Tran et al. 2020) evaluating batch correction methods recommended three methods—Harmony, LIGER, and Seurat—based on their evaluations, which we include in our analysis. Additionally, two of the most popular frameworks for manipulating scRNA-seq data sets, Seurat (Stuart et al. 2019) and SCANPY (Wolf et al. 2018), include various batch correction methods, namely BBKNN, Harmony, MNN, and Combat in SCANPY, and Harmony and SCVI in Seurat, in addition to the built-in method in Seurat. Finally, we included ComBat-seq, a newer model based on the older but established Combat method.

All of the methods evaluated are available as stand-alone libraries or part of a larger package in Python or R (R Core Team 2022).

Each method approaches the problem of batch correction in different ways. Although the algorithms and methods in the original publications follow various different workflows, we have identified common themes and shared strategies between the methods, highlighted in Table 1. The methods differ, not only in how the correction is performed, but which data object is being corrected. Namely, some methods correct the count matrix directly, whereas others leave the original matrix intact and correct either the *k*-NN graph or an embedding used to compute the *k*-NN graph.

**Table 1.** Overview of the batch correction methods

	BBKNN	Combat	ComBat-seq	Harmony	LIGER	MNN	SCVI	Seurat
Input	*k*-NN graph	Normalized count matrix	Raw count matrix	Normalized count matrix	Normalized count matrix	Normalized count matrix	Raw count matrix	Normalized count matrix
Custom embedding	None	None	None	Corrected embedding	Metagene/factor loadings	None	Learned lower dimensional latent space	CCA
Correction object	*k*-NN graph	Count matrix	Count matrix	Embedding	Embedding	Count matrix	Embedding	Embedding
Correction method	UMAP on merged neighborhood graph	Empirical Bayes—linear correction method on the count values	Negative binomial regression model on each gene	Soft *k*-means—linear batch correction within small clusters in the embedded space	Quantile alignment of factor loadings	Mutual nearest neighbors—linear correction	Variational autoencoder—models the batch effect in a low dimensional space using a deep learning model; a new count matrix is imputed from the model	Aligning canonical basis vectors to correct the embedding
Returns	Corrected *k*-NN graph	Corrected count matrix	Corrected count matrix	Corrected embedding	Corrected embedding	Corrected count matrix	Corrected count matrix and corrected embedding	Corrected count matrix
Changes count matrix	No	Yes	Yes	No	No	Yes	Yes/Imputes new values	Yes

(Input) Type of data that that particular method uses as input; the method may perform additional preprocessing steps on the input object before any calculations are performed, (Custom embedding) particular lower level embedding, if any, which the data is projected onto, (Correction object) actual data object that the method uses to make corrections, (Correction method) informal description of the particular method used for batch correction, (Returns) type of object the method returns, (Changes count matrix) whether the method edits or returns a new count matrix to be used instead of the uncorrected count matrix in any subsequent steps in the workflow.

We measure the change caused by batch correction in: the count matrix, the low dimensional embedding of the count matrix, clustering, and differential expression. Not all the methods alter the data at the same stage. Combat (Johnson et al. 2007), ComBat-seq (Zhang et al. 2020), MNN (Haghverdi et al. 2018), and Seurat (Stuart et al. 2019) alter the count matrix. Thus, we can compare the altered count matrix to the ground truth and on any downstream results. Harmony (Korsunsky et al. 2019) corrects for batch effects by computing a low dimensional principal component analysis (PCA) embedding and altering the embedding of each cell with respect to batches. BBKNN (Polański et al. 2020) changes only the *k*-NN graph-based batch information. SCVI (Lopez et al. 2018) learns a low dimensional embedding of the data using a Variational Autoencoder in a deep learning framework, from which the corrected count matrices can be imputed. LIGER (Welch et al. 2019) creates a lower dimensional embedding and a combined *k*-NN graph, which is then used to modify the lower dimensional embedding to correct for batch effects.

Because Harmony, BBKNN, and LIGER do not modify the count matrix, the batch correction affects mainly the *k*-NN graph and all downstream results that rely on the *k*-NN graph, for example, clustering. The expression estimates, in the count matrix, must then be corrected for batch effects using classical methods such as covariates in a linear model. In our results, we have excluded these methods from comparisons where the explicit differences in the count matrix are computed.

### Simulation strategy

The simplest simulation we evaluate is to generate a batch label where there is none. Namely, we take a public scRNA-seq data set and randomly label each cell as coming from batch A or batch B. This information is then supplied to each method which, in turn, corrects the data set. If the batch correction methods are well calibrated, we expect them to only make minor corrections that will not influence downstream analysis to a statistically significant degree.

Rather than simulating both count matrices and the random split, we used publicly available data sets on PBMCs from humans, neural cells from a mouse, human jejunum cells, and mouse heart cells (Methods). The data was processed using standard preprocessing workflows (Methods) to obtain the cell by gene count matrix.

**Figure 1.**
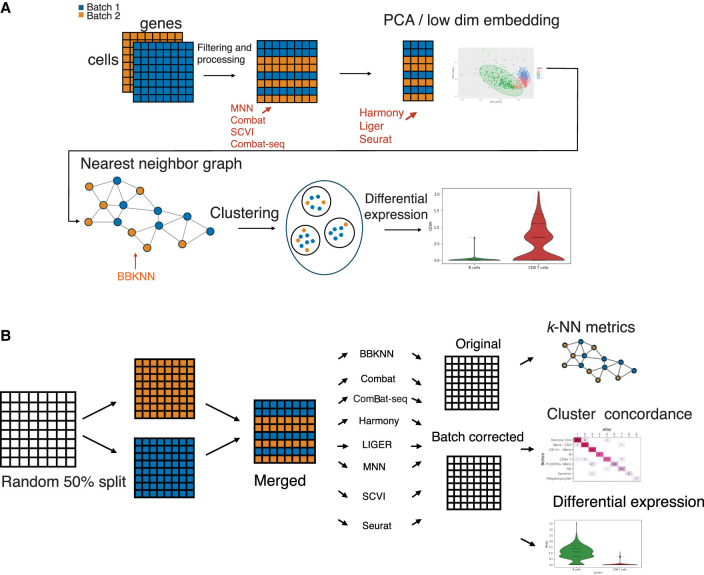
General scRNA-seq workflow using batches and the simulation strategy and evaluation in this paper. (*A*) The fundamental steps of the batch correction workflow. Data is ingested in samples, combined, and preprocessed in various ways, depending on the method. The data is then commonly projected onto a custom embedded space, possibly with lower dimension than the original data, where some correction takes place; correction can also be performed on the original count data or *k*-NN graph. The corrected object is then returned, depending on the method, a count matrix, lower dimensional embedding, NN graph, etc. Downstream methods then use this corrected object. (*B*) The general workflow of the evaluation conducted in this paper. scRNA-seq data is split randomly into two batches. Batch correction methods are applied to these two pseudobatches. The change in the data, at different stages of the scRNA-seq workflow pipeline, is then measured to assess the change that the act of batch correction has had on the data.

To do this, we created random pseudobatches for each data set, repeated 25 times (Fig. 1A). For each iteration, we performed the standard preprocessing (Methods) for the unaltered data and randomly split the cells into two batches of equal size. We then applied the batch correction methods to the uncorrected count matrices, with the pseudobatches as input. We then proceeded with the workflow, computing principal components, the *k*-NN graph, Leiden clustering, and differential expression (Fig. 1B; Methods). For each step, we compared the computed artifact to their companion in the uncorrected data and report median values across the 25 iterations.

### Changes in cell type clusters

**Figure 2.**
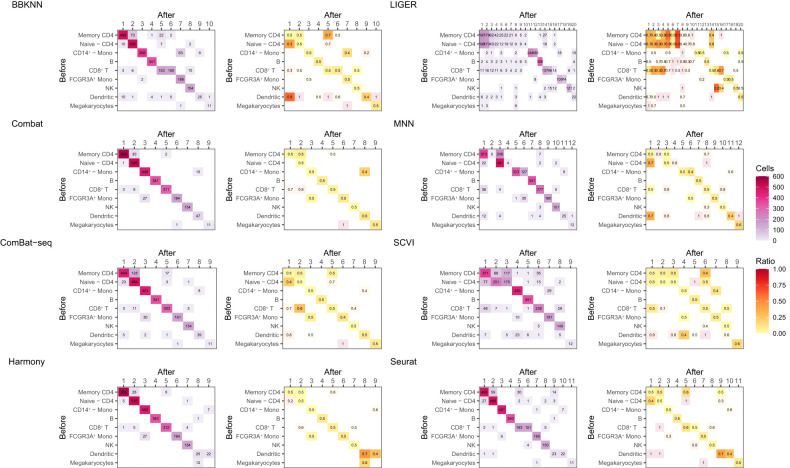
Confusion matrix of Leiden cell type clustering for the PBMC3K data. On the *left*, the squares are colored by number of cells. On the *right*, the same clusters are colored by the ratio of cells in that cluster, from batch 1, with the color intensity lowered if a square contains fewer than 10 cells. If the ratio is high or low, this would indicate that this is an artifact of batch correction as, by experimental design, each cluster should have an equal ratio of cells from batch 1 and batch 2. On the *y*-axis are the cell types before batch correction and on the *x*-axis are the cell types after. The best case result here is: (1) only values on the diagonal, where, after correction, cells in a certain cell type cluster remain with the cells they shared a cluster with; and for all ratios on the graph on the *right* to be 0.5.

The batch corrections performed can influence not only gene expression but the cluster assignment of each cell. To investigate the effects, we annotated the clusters of the uncorrected data set (Methods; Supplemental Material) and then matched the clusters obtained after batch correction. Figure 2 shows the cluster confusion matrix and the imbalance of batches within clusters for each correction method for a single iteration of the PBMC3K data. In many cases, the corrected data were split into more clusters. In those cases, we manually ordered the clusters to make the display as diagonal as possible.

We found that Harmony, Combat, and Seurat showed the least amount of cluster confusion, and Harmony and Combat performed better on the imbalance between batches. BBKNN, ComBat-seq, and MNN created more clusters, and some clusters showed high imbalance, indicating an enrichment of batch effect, creating a new cluster. On the other hand, SCVI and LIGER created the largest change in cluster number and composition with several of the clusters created being clear batch effects. The methods showed the same patterns of number of clusters and imbalance as was seen in the mouse brain data (Supplemental Figs. 5–8).

Furthermore, we saw that the relative ordering of performance was supported by computing the Adjusted Rand Index (ARI), comparing the clusters generated on uncorrected and corrected data (Supplemental Tables 1, 2). The methods generally fell into three tiers. Combat and Harmony consistently outperformed other methods on every single data set that was examined, with an ARI of 0.93 and 0.92 on the PBMC3K data, and both scoring 0.78 on the mouse brain data. Seurat performed slightly below those two with a score of 0.82 and 0.71 on the PBMC3K and mouse brain data, respectively. Finally, the remaining methods had considerably lower ARI scores on all data sets. This relative ordering was replicated in the heart, PBMC4K, and jejunum data sets and remained the same when the clusters were compared using a consensus clustering metric (Supplemental Material; Supplemental Tables 3, 4).

A simulated baseline method was also included for the heart and PBMC4K data, that consisted of simply downsampling cell reads in one batch by fifty percent. This provided a baseline of how the data would change if half of the sequenced reads in one batch are lost. Only Combat and Harmony performed better than this baseline and altered the data less than downsampling. Seurat performed better than downsampling on the heart data. Detailed cluster similarity measures can be found in Supplemental Tables 1–4.

### Differential expression

A common part of the single-cell RNA-seq workflow is finding those genes that are differentially expressed in some condition or state (Luecken and Theis 2019). One of the aims is to identify the marker genes that characterize clusters that can then be used to identify one or more cell types that are represented in the cluster. We performed differential expression using MAST (Finak et al. 2015) to identify differentially expressed genes at a Bonferroni corrected α value of 0.05.

In order to estimate the effect of batch correction on gene expression, we examined the change in the differential expression after applying each method and estimated the degree of change using the number of differentially expressed genes between two cell type clusters, before and after batch correction. We performed this comparison using two models, one with only the cluster type as a covariate and one model with cluster and batch as covariates in MAST.

We compared the methods using the PBMC3K data, the mouse brain data, and the jejunum data (Supplemental Fig. 11). Additionally, to establish a positive control for this comparison, we simulated a true batch effect by reducing the expression of the top 100 differentially expressed genes within a cluster for one of the batches.

**Figure 3.**
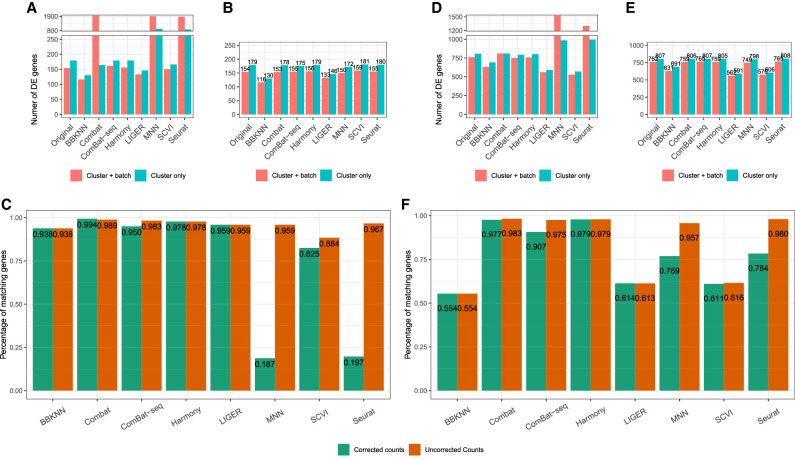
Differential expression results for the PBMC3K and mouse brain data. (*A*) Number of statistically significant genes, out of the 2000 most variable, in the PBMC3K data, the best result here being those closest to the unaltered original data. (*B*) Number of statistically significant genes, out of the 2000 most variable, in the PBMC3K data when using the original uncorrected count data but with clusters calculated on the corrected count data. (*C*) Percentage of the statistically different genes after correction in the PBMC3K data that are also found in uncorrected data for the cluster only model. The color indicates which count matrix was used for clustering. For uncorrected counts, the uncorrected count matrix was used for the model testing but with cell type clusters calculated on the corrected count matrix. A lower ratio indicates that the process of batch correction alters the genetic expression profiles of clusters. *D*–*F*, respectively, are the same as *A*–*C* but created using the mouse brain data.

For PBMC3K, we found differentially expressed genes between B cells and CD8^+^ T cells; in the original data set, this resulted in 179 and 154 differentially expressed genes using cluster, or cluster and batch, as a covariate, respectively (Fig. 3A). MNN and Seurat reported over 800 differentially expressed genes, indicating that the batch correction leaves a statistically significant trace on the gene expression that will result in too many false positives. Combat found an unusual number of statistically significant genes when correcting for the batch covariate, and this turned out to be driven by the fact that Combat performs batch correction independent of clusters or the *k*-NN graph. Overall, SCVI found a similar number of differentially expressed genes but had a lower overlap with the results from the original data set. BBKNN recovered 90% of the differentially expressed genes, but Harmony had the best performance overall.

The number of differentially expressed genes (DE) between the two cell clusters in the mouse brain data was 762 for the cluster and batch model and 807 for the cluster only model. MNN, Seurat, and SCVI performed better on the mouse brain data compared to the PBMC3K data—we saw similar spikes for the cluster and batch model for MNN and Seurat, but the absolute difference in number of genes was much less here than in the PBMC3K data. BBKNN and LIGER performed worse, at around 200 genes less than the uncorrected data. For all the methods tested, we saw an improvement when using the clustering created on the uncorrected data.

When looking at the percentage of genes that are differentially expressed in the original data and the corrected data in Figure 3, C and F, we see that just over half of the DE genes in the BBKNN, LIGER, and SCVI corrected data are also DE in the uncorrected data in the mouse brain data. This is a decline in performance that was not observed in the PBMC3K data analysis, in Figure 3C. Although the number of DE genes is consistent with the uncorrected data, for BBKNN, LIGER, and SCVI, the overlap of DE genes is less than half.

The methods performed similarly on the jejunum data (Supplemental Fig. 11) as they did on the PBMC3K, when comparing the differential gene expression between tuft cells and goblet cells. The number of differentially expressed genes increased by a similar factor—from around 200 to around 1900—when correcting with Combat, MNN, and Seurat using the cluster and batch MAST model.

On all three data sets, Harmony had the best performance and resulted in the least amount of change of DE genes.

To isolate the differences between the results on the original data set and the batch-corrected versions, we repeated the differential expression analyses but used the uncorrected count matrices and the cluster assignments from the batch-corrected data sets. Comparing panels A and B for the PBMC3K data set and panels D and E for the mouse brain data set in Figure 3, we see that the poor performance of MNN, Seurat, and Combat is driven by the modifications of the gene expression counts rather than solely the cluster assignments, whereas Harmony, Liger, and BBKNN do not adjust the count matrix.

We repeated the analysis on the PBMC3K and mouse brain data sets with batch effects simulated and identified the same patterns as in the absence of batch effects (Supplemental Figs. 12, 13).

**Figure 4.**
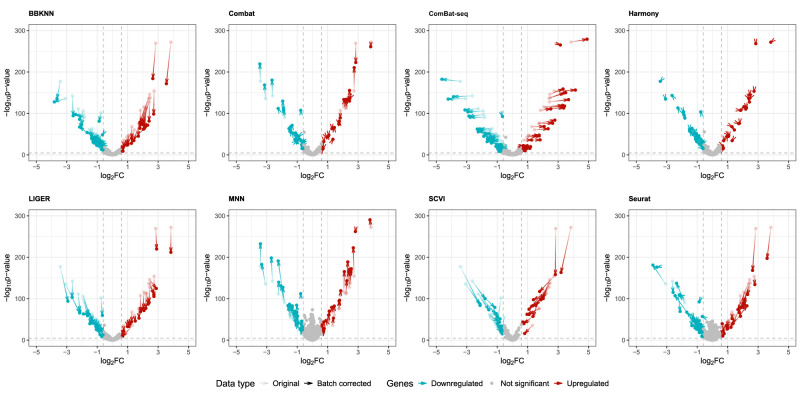
The volcano plot for each method using the same MAST model (cluster only). Using the comparison as in Figure 3, differentially expressed genes when comparing B cells and CD8^+^ T cells, for the PBMC3K data. The genes have been colored depending on if they are upregulated or downregulated. The horizontal dashed line is the Bonferroni-adjusted *P*-value cutoff at 0.05 level of significance. The vertical dashed lines signify a log_2_ fold change of 0.6. The genes *above* the horizontal dashed line and to the *right* or *left* of the vertical dashed line are marked as up- or downregulated. Gray points are genes that are not deemed significant or expressed differentially. Genes are only included if a valid result was returned by the MAST model. For the up- or downregulated points in each figure, there are arrows drawn from the point where a particular gene lands on the plot in the uncorrected data and ends at the point where the gene is in the corrected data. An optimal batch correction would have very short lines between points, indicating a low degree of change.

In addition to the difference in the number of differentially expressed genes in Figure 3, we assessed the degree of difference between the genes in the two cell type clusters in Figure 4. The figure includes a volcano plot for each method using the batch only model in Figure 3. This was consistent with the results in Figures 2 and 3—Combat and Harmony cause the smallest degree of change in the data. ComBat-seq does not inflate *P*-values, but it increases upregulated genes and reduces downregulated genes. MNN, BBKNN, and LIGER appear to have consistent estimates and log_2_ fold change, but we see general inflation of *P*-values for MNN and a deflation for LIGER and BBKNN. SCVI shows a decrease in both log_2_ fold change and *P*-values, indicating an overall loss in differentially expressed gene signal between the two cell clusters. As is consistent with our other metrics, Harmony creates the smallest effect on the *P*-values and log_2_ fold change out of all the methods.

When examining the genes that are not up- or downregulated, we split them into two categories: high signal—those that are statistically significant but are below the threshold for log_2_ fold change; and low signal—those that are below the threshold for both log_2_ fold change and *P*-values. What we can see is that some methods affect the gene expression for most of the genes in the clusters. MNN and Seurat reduce signal; that is, the difference in expression that exists between the clusters is systematically reduced for high signal genes (Supplemental Figs. 15–18).

### Nearest neighbor rank—embeddings

The *k*-NN graph is a fundamental object that is used as input in several widely used algorithms in single-cell RNA-seq data analysis, for example, graph-based clustering (Leiden) and the creation of UMAP figures. Thus, we wanted to measure the extent of the change in the neighborhood composition of cells created by the process of batch correction.

**Figure 5.**
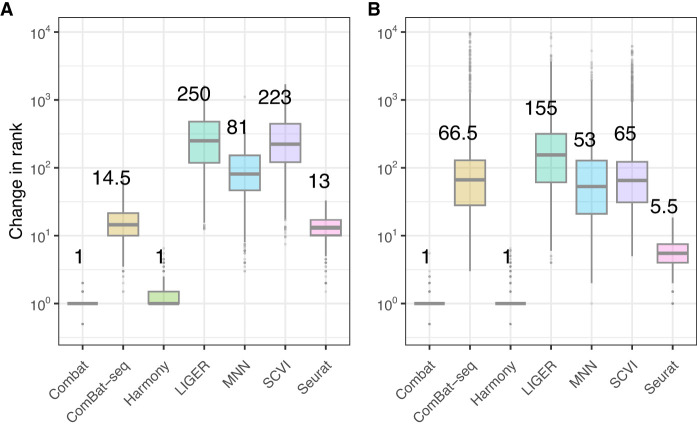
Change in NN rank for the PBMC3K and mouse brain data. (*A*) The PBMC3K data. (*B*) Mouse brain data. Median change in absolute rank difference for the top 30 nearest neighbors of each cell for each method in the lower dimensional embedding, after batch correction.

Comparing the rank displacement in the lower dimensional embedding in Figure 5, A and B, we see that Combat and Harmony consistently have the smallest rank displacement, whereas SCVI, Liger, and MNN have the highest displacement, representing, on average, no overlap among the top 30 neighbors.

These comparisons use dimensionality reduced data, top 50 PCA components for count-based methods (Combat, MNN, Seurat), and corrected embedding (Harmony, Liger, SCVI), whereas BBKNN was left out because it alters the *k*-NN graph but not the distances required to order neighbors. We observe a similar performance of the count-based methods when the rank displacement is computed on the full count matrix rather than a lower dimensional embedding (Supplemental Figs. 1, 2). Because Harmony uses the PCA components to compute distances, and we used the PCA to define the ground truth, we tested the effect of limiting the number of PCA components shown to Harmony to measure the effect of having less information to work with and found that the performance was not sensitive to the distance estimates (Supplemental Fig. 14).

Additional NN results for the embeddings and counts for the PBMC4K, heart, and jejunum data can be found in the Supplemental Material (Supplemental Figs. 2–4).

## Discussion

We tested eight methods for batch correction and by constructing randomized pseudobatches, we examined the degree of change in various metrics, before and after batch correction.

Overall, we see that the methods tend to fall into three categories of performance. Combat and Harmony performed the best or close to it in all the evaluations we performed. Those two methods introduced the fewest artifacts into the data during the process of batch correction. Seurat introduced artifacts that are more easily identifiable but seems to retain the overall structure of data when comparing clusters. Finally, ComBat-seq, MNN, SCVI, LIGER, and BBKNN all introduced significant changes that altered local neighborhood structure, clusters, and differential expression results.

Although Combat performed well in the tests performed in this paper, it has been found to be outperformed by more modern methods when it comes to removing batch effects (Haghverdi et al. 2018; Tran et al. 2020). Harmony is well suited for a variety of integration with a low number of artifacts being introduced, in cases where there is little batch difference. We note that the version of Harmony benchmarked here (v0.1) is not the most recent, but we did not detect any significant changes when running v1.0 on a small subset of the data, as there were no fundamental changes to the algorithm.

It is an interesting problem to pinpoint exactly why the various methods tend to overcorrect the effects of batches. As shown in Table 1, the methods employed are diverse enough that no central theme emerged. In reviewing the description of the methods, we noticed that Harmony was the only method that tried to tone down the correction if the batches were not biased locally. To test this, we varied some of the parameters in Harmony to try to turn off this behavior, which resulted in worse performance (Supplemental Figs. 18, 19). This explains, in part, why Harmony is resistant to overcorrection in the presence of no batch effects. Additionally, we inspected the role the preprocessing might have played and found no effect of using Seurat rather than SCANPY for preprocessing (Supplemental Fig. 20).

The role of batch correction in single-cell RNA-seq analysis is twofold. First, we want to account for batch effect, so that cells of the same cell type are clustered together in the combined data set. Second, after forming clusters, we need to account for the batch effect in other downstream methods (e.g., differential expression or finding marker genes).

The count-based methods, MNN, SCVI, Seurat, ComBat-seq, and Combat, aim to solve both problems by altering the count matrix, whereas the other methods only tackle the first issue. Based on our results, we can only recommend Harmony for general purposes because it performs the best for all metrics we examined. Although Harmony does not correct the count matrix, the batch covariate can be used as input in statistical methods (e.g., MAST) to account for the batch effects.

In general, we find that methods that correct local structure for batch effects (i.e., by computing an embedding or *k*-NN graph used for clustering) perform better than methods that aim to correct the count data directly. We find significant artifacts introduced in differential expression results in methods that correct the count data. This effect is reduced when the corrected expression is only used for clustering but the statistical tests for differential expression are run on the uncorrected data. Although it is convenient to fix the counts once and for all, we advise future method developers to focus on methods that correct for batch effects on the clustering step or the generation of *k*-NN graphs and to use well-studied classical methods when accounting for batch effects in statistical tests such as differential expression.

## Methods

### Simulated data

We simulated a difference in expression level for a particular cell type by performing ad hoc clustering and reducing the UMI counts by 50%, before any normalization or correction, for the top 100 most differentially expressed genes. The clusters modified were microglial cells in the mouse brain data set and in B cells in the PBMC3K data set.

### Analysis workflow

For all the data sets we used SCANPY (Wolf et al. 2018) to perform basic preprocessing. In addition to filtering out cells with few detected genes and genes detected in few cells, cells with a high fraction of mitochondrial counts were also removed. Library size normalization was then performed before log-transformation. In the case of the mouse brain data, in addition to this, the data were scaled and variation was regressed out.

To standardize the comparison, the same 2000 highly variable genes, calculated on the uncorrected data, were used as input except in the case of the alternative workflows of LIGER and Seurat, where we find the most variable genes using methods provided in the libraries themselves, as is recommended in these studies (Butler et al. 2018; Welch et al. 2019). This alternative comparison is presented in Supplemental Figures 9 and 10. Where possible, preprocessing was kept identical for each method (Supplemental Fig. 21). A detailed discussion of the preprocessing can be found in the Supplemental Material.

A *k*-NN graph was created using the PCA embedding using SCANPY with default values. Following that, cell clusters were calculated using the Leiden algorithm with default settings, using SCANPY (Traag et al. 2019).

Each step in the workflow was run in SCANPY or a Python library if possible. For those batch correction methods that only provide libraries or are more commonly implemented in R, the preprocessing was performed in Python, then the batch correction was run in R using Seurat where possible. All the data sets were kept in the Anndata format in Python (Virshup et al. 2023). The data were exported to files and imported in R when required.

### Downsampling

To estimate a baseline of technical variation to expect when integrating data sets with different modalities, we compared our results to simulated batch effects with downsampling. Our aim was to provide a lower bound of change in our evaluation criteria. We wanted to evaluate if the batch correction methods alter the data more than simply downsampling one batch and using the combined data without additional correction. For the heart and PBMC4K FASTQ files, we downsampled the data using BUStools (Melsted et al. 2019). For both sets of data, we downsampled the reads of all cells within one batch to 50%. To standardize these results, we used only cells found in one of the pseudobatches in the original uncorrected data set. There was, therefore, no cell filtering performed on the Anndata file created using the downsampled BUS files.

For each method, there exists a plethora of different configurations and parameters that can be configured to adapt the method to different scenarios. In this case, the default parameters were used whenever possible. In some cases, we selected parameters to enable easier comparison between the different methods, for example, selecting the top 2000 most variable genes instead of using the default method in SCANPY. This ensured that for each method the number of highly variable genes was the same. Additionally, whenever possible we provided a seed to make a randomized method return deterministic results.

### Data used in this paper

Publicly available data sets from 10× Genomics were used for analysis and simulations (Supplemental Material). The PBMC3K data was derived from peripheral blood mononuclear cells from a healthy donor, generated by 10× Genomics, with a sequencing depth of 69,000 reads per cell. The mouse brain data set was derived from brain tissue of two E18 mice, with around 18,500 reads per cell. We used a 20-k cell sample provided by 10× Genomics. The PBMC4K data set is similar to the PBMC3K data set, except the sequencing depth is higher at around 87,000 reads per cell. The mouse heart data was derived from the whole heart of a mouse, with a sequencing depth of around 83,000 reads per cell.

The PBMC4K data and heart data were processed from the original FASTQ files. This enabled us to perform additional comparisons using downsampling. All other data were read using raw H5 or MTX files. An additional reason to include the PBMC4K data was for its very high read depth, when compared to the other data sets.

### Evaluation criteria

#### Change in nearest neighbor rank

Neighborhood structure is an important part of downstream analysis in scRNA-seq. The neighborhood of different cells allows us to get an understanding of the local structure. Commonly, a *k*-NN graph is created to define the *k*-nearest neighbors of each cell for all cells. An important downstream result that depends on the *k*-NN graph is the cluster analysis. The commonly used Leiden (Traag et al. 2019) method uses the NN graph to perform clustering. In each case, the *k*-NN graph was calculated using the default parameters in SCANPY.

**Figure 6.**
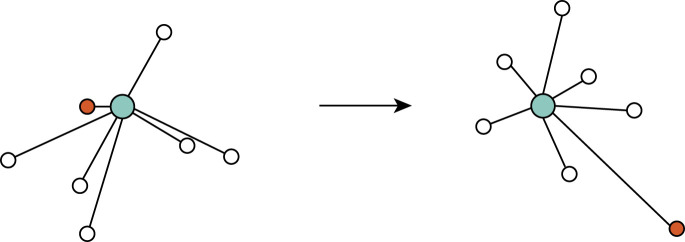
Visual representation of the NN rank comparison. Given a NN graph of the center cell *c,* in blue, before and after batch correction. Before batch correction, the orange cell has a rank of 1; that is, it is the closest neighbor to cell *c*. After batch correction, it has a rank of 7. We define rank displacement as the difference between these values in NN rank.

In order to measure the effect that the batch correction methods have on the nearest neighbor structure, we examined the location or rank, in terms of order of closest to furthest, of nearest neighbors of each cell before and after batch correction (Fig. 6). For those methods that change the count matrix, we used the log-normalized count matrix to calculate the change in NN rank. The neighborhood structure is commonly determined in some lower dimensional space. In order to get a concise measure of the extent of the nearest neighbor displacement, we additionally examined the change in nearest neighbor structure in the lower dimensional embedding. We compared the NN rank on the PCA embedding on the uncorrected data to a PCA embedding created on the corrected count matrices, except in the case of Harmony, LIGER, and SCVI, which return a corrected embedding that is used for comparison.

We calculate the following for each cell *i* and nearest neighbor (NN) *j*:

$$med_{30}(abs(rank(NN_{ij})-rank(NN_{ij}^{*}))),$$

where *med*_30_ is the median over the top 30 nearest neighbors, and $rank(NN_{ij}^{*})$ is the rank of the *j*th NN of cell *i* after batch correction.

#### Differential expression

To assess if batch correction methods have an effect on differential expression results, we compared the number of statistically different genes between two distinct cell clusters in the data, before and after correction. As we only tested the top 2000 most variable genes, the maximum number of differentially expressed genes would be 2000. For the PBMC3K data set, we compared clusters that represented B cells and CD8^+^ T cells. For the mouse brain data, we considered smooth muscle cells and microglial cells. For the jejunum data, we selected tuft cells and goblet cells. These cell types were chosen because they have highly differentially expressed, unique markers (Tasic et al. 2016; Haber et al. 2017; Li et al. 2022). The clusters used were identified by first using Leiden unsupervised clustering, followed by manual identification of the cluster corresponding to the cell type by ranking marker genes. For each iteration, we identified the clusters that had *MS4A1* as a top-200 marker gene and labeled these as B cells. We did the same for *CD8A* and CD8^+^ T cells. Similarly, for the mouse brain and jejunum data, we differentiated the clusters based on the unique cell type markers.

We used two different MAST (Finak et al. 2015) models, and each model was then run for each gene. We used as input into the model only the cells in the clusters being compared. The MAST model is a two-part hurdle model that models the rate and level of expression for cells for each gene, which allows for the controlling of covariates. We included batch and cluster as covariates to assess the statistical significance of each on gene expression. The models we implemented are as follows:

Y∼cluster,

Y∼batch+cluster.

A likelihood-ratio test (LRT) was then performed, using the MAST software package, to assess the significance of the covariate in question. The *P*-values for the cluster covariate in the cluster only model, and both covariates in the batch and cluster model, were computed and the cutoff for significance selected was the Bonferroni-adjusted *P*-value originally at 0.05 significance. An LRT was performed for each gene.

We used, as a ground truth, the genes determined to be differentially expressed in the original data set without any batch correction. For each batch correction method, we determined the differentially expressed genes between the two clusters. The clusters used in the analysis were chosen as the two clusters that have the same particular marker genes as the clusters in the original data set. We also performed the differential expression analysis using the exact set of cells as in the analysis without batch correction.

As an additional comparison of the effect of correcting the original count matrix, we ran the same models using MAST, but instead of using the corrected count matrix, we used the corrected cluster assignments provided by the methods or calculated after using the methods. However, the count matrix contains the uncorrected original counts.

A separate analysis of the degree of difference in gene expression is presented in the form of a volcano plot. These plots consist of negative log_10-_transformed *P*-value on the *y* axis and the log_2_ fold change on the *x* axis. The log_2_ fold change was calculated using the *getLogFC* function in the MAST software package. The *P*-value was calculated using the output of the hurdle model in MAST.

### Software availability

All code used to perform the analysis is available at GitHub (https://github.com/pmelsted/AM_2024) and as Supplemental Code.

## Supplemental Material

Supplement 1
